# Three-dimensional evaluation using CBCT of the mandibular asymmetry and the compensation mechanism in a growing patient: A case report

**DOI:** 10.3389/fpubh.2022.921413

**Published:** 2022-11-01

**Authors:** Monica Macrì, Felice Festa

**Affiliations:** Department of Innovative Technologies in Medicine and Dentistry, University “G. D'Annunzio” of Chieti-Pescara, Chieti, Italy

**Keywords:** facial asymmetry, dental midline deviation, CBCT, rapid palatal expander, Fränkel-III, orthopedic therapy, orthodontic therapy

## Abstract

**Background:**

This case report aims to evaluate the development and the compensation mechanisms of the mandibular asymmetry in a growing male patient using cone beam computed tomography (CBCT). In this case, the menton deviated on the right, a sporadic condition, which may be the consequence of a disorder in the mandibular growth.

**Case presentation:**

The young male patient was treated with rapid palatal expander (RPE) and Fränkel functional regulator III (FR-3). The initial CBCT was acquired at the beginning of therapy when the patient was 8 years old, and the final CBCT was developed at the end of the treatment when the patient was 12 years old. The patient's CBCT was performed with the head oriented according to the Natural Head Position (NHP); the NHP is a physiological and reproducible posture defined for morphological analysis. The 3D image of the cranium was oriented in the Dolphin software according to NHP posture, and cephalometric measurements were taken in the software's frontal, laterolateral right and left, posteroanterior, and submentovertex views. The therapy lasted 3.8 years and ended with significant regression of the mandibular asymmetry from moderate grade (4.2 mm) to slight grade (1.3 mm).

**Conclusion:**

The literature shows that the left hemi-mandible has grown more than the right side, which affirms that in case of deviation of the menton >4 mm, the bone volume increases on the non-deviated side.

## Background

*Facial asymmetry* is the difference in shape, size, position, or function between the two sides of the face (1). In most cases, the asymmetry is not clinically detectable; it is also known as subclinical, minor, or normal facial asymmetry (2).

A dominant half-face is recognized in all subjects: in 80% of cases, it corresponds to the right side, with no differences in distribution according to sex and age (3). The dominance of the right side is explained by the migration of the cells of the cranial neural crest (NCC): migration begins earlier on the right side than on the left side, but it ends simultaneously on both sides; for this reason, there is an evident dominance on the right side of the face; consequently, the menton left shift (the most inferior point on mandibular symphysis) is more frequent than the right shift (4).

In addition, mandibular asymmetry is more frequent than maxillary asymmetry. The growth of the maxilla is more stable due to the connection with the cranial base synchondroses, and it is less vulnerable to environmental factors influence; differently, the mandible is the only mobile bone in the skull, and for this reason, it is highly prone to environmental impacts (5).

The right shift of the menton is a rare condition that may result from a disorder in the mandibular growth (i.e., facial trauma, TMJ ankylosis, bad habits, prone sleep position, premature tooth loss, and iatrogenic causes) (6).

The craniofacial growth can be compromised if a pathogenic noxa affects an evolutionary age, producing deformities and asymmetries in the head–neck district.

It is essential to detect dentofacial asymmetries in orthodontic practice: the dental midline is a reference landmark that must coincide with the center of the mouth (the imaginary line that joins the center of the philtrum with the center of the palatine raphe). The mandibular midline corresponds to the inferior interincisal line (7).

When a clinician observes a mandibular asymmetry in children, he has to think of a functional asymmetry, which must be corrected to prevent its transformation into a skeletal and joint asymmetry. Using the Frankel function regulator, it is possible to re-center the two arches and restore muscle function, breathing and vocalization. If a mandibular asymmetry is detected within 6 years of age, it can be fully recovered, preventing TMD (8) and joint problems in future adult patients (9).

Treating mandibular asymmetry as soon as it is detected is important, and it has practical results if treated during primary dentition. Frankel's function regulator type 3 is very effective, especially in treating third-class malocclusions, even if treated in early mixed dentition (10).

With its particular shape and design, the device promotes maxillary growth by retracting soft tissues that block it and stimulating the periosteum, directing mandibular growth (11). The device consists of four resin shields: two on the anterior part and the other on the sides. The upper anterior shields eliminate the pressure of the upper lip on the underdeveloped jaw. The two vestibular shields act superiorly by stimulating the periosteum and relieving the pressure of the buccinator (12).

Controlled retrospective studies show that the craniofacial changes following the treatment with Frankel-III are stable. There is no significant inhibition of mandibular growth but the closure of the gonial angle. Intermaxillary and interdental changes are maintained and stable over time (13).

Some authors recommended that to be effective, long-term appliance wear (more than 5 years) is necessary to achieve clinically valuable results in FR-3 appliances (14).

The present case report describes the successful orthopedic and orthodontic treatments of an 8-year-old Caucasian patient with an anterior crossbite and severe mandibular deviation to the right side.

The orthopedic–orthodontic treatment lasted 3.8 years and was divided into two phases: the first phase with the RPE and the second phase with the FR-3. The patient was 8 years at the beginning of therapy and 12 years at the end. The CBCT scans were acquired at the treatment's beginning (T0) and the end (T1).

## Case presentation

### Diagnosis and etiology

An 8-year-old male patient visited the Orthodontic Department at G. D'Annunzio University in Chieti, Italy, with a chief complaint of anterior crossbite and mandibular asymmetry. No systemic pathologies or maxillofacial disorders were found in the medical history.

The facial evaluation showed a straight profile and a soft-tissue asymmetry of the lower face with a mandible shift to the right side. Intraorally, the dentition was mildly crowded in the upper arch, and a class III molar relationship was observed on the left and right sides. The mandibular dental midline was deviated 4 mm to the right, whereas the upper dental midline coincided with the facial midline.

The patient exhibited a normal overbite and an anterior crossbite with a −2.0 mm overjet.

The dental cast analysis at T0 revealed a maxillary transverse deficiency: the upper arch width was 2.5 mm narrower than the lower arch in the first molar region.

The cephalometric analysis at T0 reveals a class I skeletal profile (15) (ANB: +0.9°), mesocephalic (16) (SN—GoGn: 30.1°), hypodivergent growth pattern (17) (FH—GoGn: 13.6°), and moderate right shift of the menton (4.2 mm) (18).

### Cone beam CT analysis

All CBCT examinations were taken at T0 and T1 and were performed by the Planmeca ProMax^®^ 3D MID unit (Planmeca Oy, Helsinki, Finland) according to the low-dose protocol (19) with these parameters: large FOV, standard resolution quality images, 80 kVp, 5 Ma, and acquisition time of 15 s resulted in an effective dose of 35 microsieverts (μSv) (20).

The three-dimensional graphic rendering software used for the cephalometric measurements was Dolphin Imaging 11.95 Premium (Patterson Technology, Chatsworth, CA). The software processes the 3D-CT scan images in 2D-Digital Imaging and Communications in Medicine (DICOM) files.

The patient's CBCT was performed with the head oriented according to the NHP; the patient was in a sitting position with the back perpendicular to the floor as much as possible. The head was stabilized with ear rods in the external auditory meatus. The patient was instructed to look into their eyes in a mirror 1.5 m away to obtain NHP. The NHP is a physiological and reproducible posture defined for the morphological analysis described in the orthodontic and anthropological literature (21).

The 3D image of the cranium was oriented in the Dolphin software according to NHP posture before taking cephalometric measurements.

**Figure 1:**
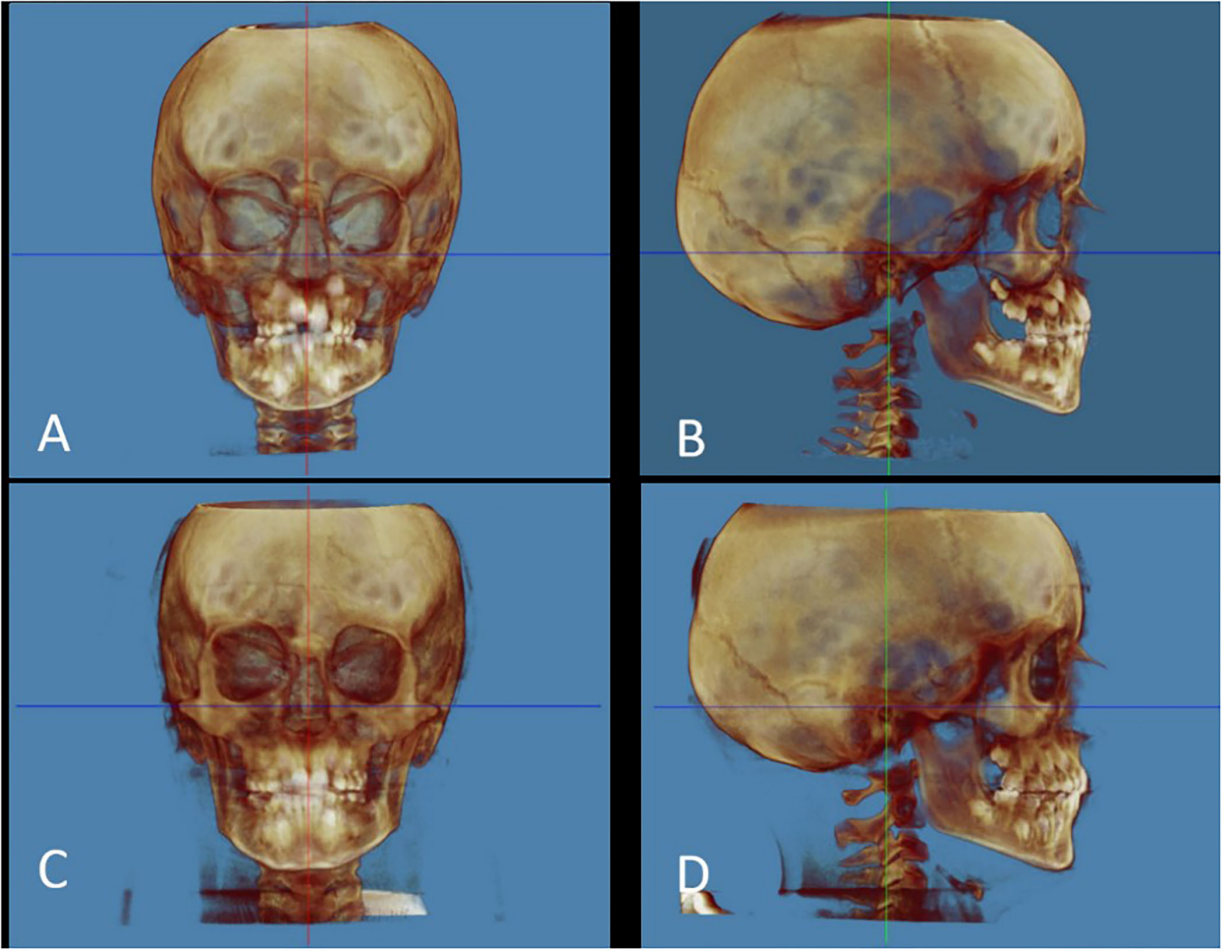
Natural head position. **(A)** Pre-treatment frontal view; **(B)** Pre-treatment lateral view (right); **(C)** Post-treatment frontal view; **(D)** Post-treatment lateral view (right). The red line corresponds to the sagittal plane. The green line corresponds to the coronal plane. The blue line corresponds to the transverse plane. The reference landmarks used for cephalometric measurements are shown in Table 1.

The NHP orientation was carried out by the widgets present in Dolphin; hard and soft tissue views were checked for orientation in the software by visualizing the head from the front, right, and left sides. In the NHP, there are three reference planes (Figure 1), perpendicular to each other, which are identified on the software for the patient's cephalometric measurements.

1. The transverse plane coincides with the Frankfurt plane (FH), a plane passing through two points: Orbital (Or) and Porion (Po);
2. The sagittal plane coincides with the mid-sagittal plane (MSP), a plane perpendicular to the plane FH and passing through two points: crista galli (Cg) and basion (Ba);
3. The coronal plane coincides with the anteroposterior (PO) plane, perpendicular to the FH and MSP, passing through the right and left Porion.

**Table 1:** Reference cephalometric landmarks.

**Landmark**	**Abbreviation**	**Description**
Crista Galli	Cg	The most superior point of the crista Galli of the ethmoid bone
Basion	Ba	The median point on the anterior margin of the foramen magnum
Porion	Po	The highest point on the roof of the external auditory meatus
Orbitale	Or	The deepest point on the infraorbital margin
Condylion superius	Cdsup	The most superior point of the condyle head
Condylion medialis	Cdmed	The most medial point of the condyle head
Condylion lateralis	Cdlat	The most lateral point of the condyle head
Condylion posterius	Cdpost	The most posterior point of the condyle head
Sigmoid notch	S	The most inferior point of the sigmoid notch
Gonion lateralis	Golat	The most lateral point of the gonion area
Gonion posterius	Gopost	The most posterior point of the gonion area
Gonion inferius	Goinf	The most inferior point of the gonion area
Menton	Me	The most inferior point on the mandibular symphysis
First maxillary molar	6	Occlusal fossa of the maxillary first molar
Mandibular canine	3	Cuspal tip of the mandibular canine

The CBCT measurements (Table 2) were performed in frontal, laterolateral (LL) right, LL left, posteroanterior (PA), and submentovertex (SMV) views. Each measurement was performed on the initial and final CBCT. Also, the size of the right and left masseter muscles was evaluated with a widget present in Dolphin. In the frontal view, the size of each muscle was measured by adjusting the translucency instrument to discriminate soft from hard tissues.

**Table 2:** Cephalometric measurements.

	**Landmarks**	**Pre-treatment**	**Post-treatment**	**Results**
**Frontal view (F)**			
Menton deviation	Distance from Me to MSP	4.2 mm (moderate deviation)	1.3 mm (slight deviation)	Δ: −2.9 mm
Right masseter muscle	Maximum length and width	Lenght: 55.4 mm	Lenght 61.5 mm	Δ: +6.1 mm
		Width: 15.7 mm	Width: 19.4 mm	Δ: +3.7 mm
Left masseter muscle	Maximum length and width	Lenght: 51.0 mm	Lenght 54.3 mm	Δ: +3.3 mm
		Width: 11.3 mm	Width: 14.9 mm	Δ: +3.6 mm
**Laterolateral view (LL)**			
Vertical facial growth pattern	Angle from SN to GoGn	30.1° (mesofacial)	32.5° (mesofacial)	Δ: +2.4°
Frankfort-mandibular plane angle (FMA)	The angle from FH to GoGn	13.6° (hypodivergent)	16.8° (hypodivergent)	Δ:+3.2°
Sagittal facial growth pattern (ANB)	The angle from A to N to B	0.9° (class I)	2.5° (class I)	Δ: +1.6°
Right–left difference in lateral Ramal inclination	The angle from Cd post—Go post to FH	Right: 74.7°	Right: 77.1°	Δ: +2.4°
		Left: 73.4 mm	Left: 71.8°	Δ: −1.6°
Right–left difference in ramus length (without condyle and gonial angle)	Distance from Copost gopost	Right: 37.7 mm	Right: 38.9 mm	Δ: +1.2 mm
		Left: 33.8 mm	Left: 41.6 mm	Δ: +7.8 mm
Right–left difference in ramus length (with condyle and gonial angle)	Distance from Cdsup to Go inf	Right: 50.9 mm	Right: 55.8 mm	Δ: +4.9 mm
		Left: 48.9 mm	Left: 54.6 mm	Δ: +5.7 mm
Right–left difference in condylar height	Distance from Cdsup to S	Right: 18.3 mm	Right: 17.3 mm	Δ:−1.0 mm
		Left: 18.1 mm	Left: 20.2 mm	Δ: +2.1 mm
**Postero-anterior view (PA)**			
Right–left difference in maxillary height	6 to FH	Right: 29.0 mm	Right: 35.8 mm	Δ: +6.8 mm
		Left: 27.2 mm	Left: 37.0 mm	Δ: +9.8 mm
Right–left difference in frontal Ramal inclination	The angle from Cdlat-Golat to MSP	Right: 20.4°	Right: 14.9°	Δ:−5.5°
		Left: 16.5°	Left: 16.9°	Δ: +0.5°
Right–left difference in mandibular body height	Distance from 3 to GoGn	Permanent canines not erupted	Right: 53.1 mm	Not evaluabe
			Left: 33.3 mm	
Intercondilar distance	Distance from right Cdmed to left Cdmed	74.0 mm	83.3 mm	Δ: +9.3 mm
Extracondilar distance	Distance from right Cdlat to left Cdlat	102.7 mm	107.9 mm	Δ: +5.2 mm
Maximum width of the left condyle	Distance from Cdlat to Cdmed	15.0 mm	16.1 mm	Δ: +1.1 mm
Maximum width of the right condyle	Distance from Cdlat to Cdmed	15.1 mm	16.5 mm	Δ: +1.4 mm
Right–left difference in condyle—MSP distance	Distance from Cdlat to MSP	Right: 50.3 mm	Right: 51.7 mm	Δ: +1.4 mm
		Left: 52.3 mm	Left: 53.8 mm	Δ: +1.5 mm
**Sub-mentovertex view (SMV)**			
Right–left difference in mandibular body length	Me-Gopost,	Right: 76.4 mm	Right: 77.4 mm	Δ: +1 mm
		Left: 74.9 mm	Left: 82.4 mm	Δ: +7.5 mm

Δ Difference (post-treatment data – pre-treatment data), FH, Frankfort Horizontal plane; PO, anteroposterior reference plane; MSP, mid-sagittal reference plane; GoGn, mandibular plane.

### Treatment objectives

Based on the clinical and radiographic findings, the primary objectives of treatment were planned as follows: (1) correction of the dental and skeletal mandibular midlines, (2) correction of the dental class III malocclusion, (3) correction of the anterior crossbite, (4) making space on the maxillary dentition for guiding eruption and correction of the mild crowding, and (5) correction of the negative overjet.

### Treatment alternatives

Option 1. The orthopedic–orthodontic treatment with RPE and FR-3 was proposed as the first-choice treatment based on the treatment objectives.

Option 2. The orthopedic treatment with a class III protraction facemask was proposed as an alternative treatment.

Option 3. If orthopedic–orthodontic treatment (options 1 and 2) could not be performed, orthognathic surgery could be a choice after completing skeletal growth. However, option 3 was poorly recommended because of the surgical risks and costs of surgical intervention, whereas option one was highly recommended and chosen with the consent of the patient's parents.

### Treatment progress

The orthopedic therapy was performed in two phases: the first phase with a rapid palatal expander (RPE) and the second phase with the Fränkel function regulator III (FR-3).

The first phase of the treatment uses the RPE, which provides a transverse expansion of the maxilla; the RPE was initially activated on the chair by performing a complete turn of the screw, which corresponds to four activations (1 mm). The patient was instructed to activate the RPE at home two times daily (0.5 mm expansion a day) for 10 days. The same RPE was used as a passive retainer to prevent transverse maxillary relapse for 6 months, and the screw was locked with a light-cure flow composite. The appliance was removed after 6 months after its last activation. The second phase with the FR-3 corrected skeletal deformities and prognathism. The therapeutic principle is based on eliminating all factors that could arrest maxillary development and, at the same time, prevent excessive mandibular growth (19).

### Treatment results

The facial evaluation showed an improved soft-tissue symmetry in the lower face. Intraorally, ideal occlusion, proper overjet, and I molar relationship were achieved. The dental cast analysis revealed the achieving of proper maxillary and mandibular intermolar widths and revealed a partial re-centring of the mandibular midline was achieved (2.9 mm to the left), as confirmed by CBCT; however, at the end of the therapy, the menton still deviated 1.2 mm to the right (slight deviation) (22). The CBCT cephalometric analysis before and after the treatment is shown in Table 2.

As described in the literature (23), the menton point is the most inferior point on mandibular symphysis in the median plane. In this case report, the mandibular deviation was evaluated, calculating the deviation of the menton from the MSP. At T0, the menton deviation was 4.2 mm (moderate deviation) and after the treatment was 1.3 mm (slight deviation).

After the treatment, the menton point moved 2,9 mm toward the reference midline.

**Figure 2:**
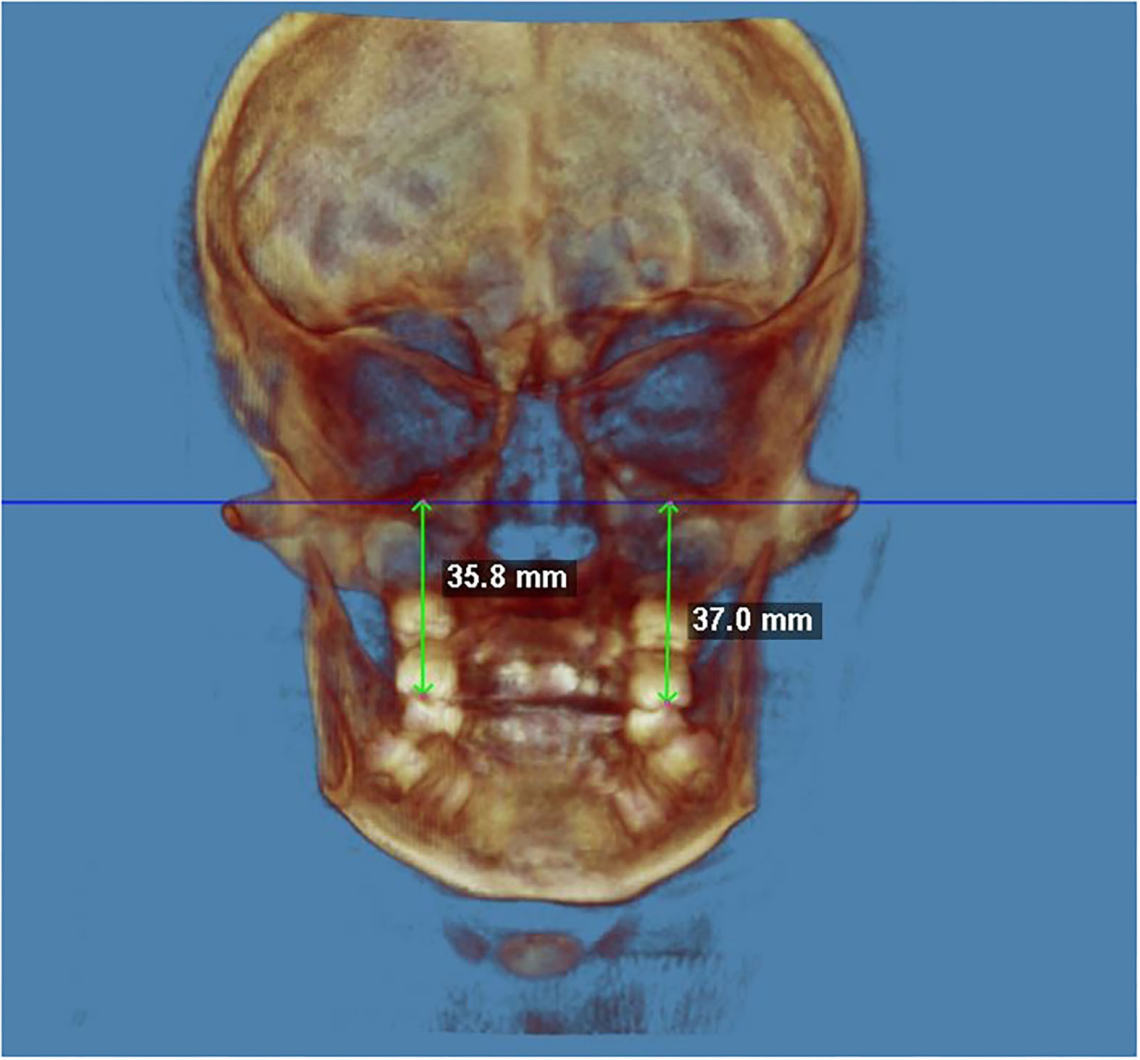
The right–left difference in maxillary height at the end of the treatment (PA view). The maxillary height was calculated from FH to the occlusal fossa of the maxillary first molar.

The cephalometric analysis of the masseter muscles (Figure 2) showed that both muscles developed similarly in thickness but not in length. The maximum length of the right masseter muscle was 55.4 mm at t0, and 61.5 mm at t1, with a difference of +6.1 mm. The maximum length of the left masseter muscle was 51.0 mm at t0, and 54.3 mm at t1, with a difference of +3.3 mm. The length of the right muscle has increased more than the left muscle, and this result positively affected the re-centring of the menton points toward the MSP. This finding is significant because it was shown that if mandibular asymmetry is not corrected, the mandible may grow and develop asymmetrically due to lateral displacement and asymmetric muscle function.

On the laterolateral view (LL), the cephalometric analysis evaluated the vertical facial growth pattern, the Frankfort-mandibular plane angle (FMA), the Sagittal facial growth pattern (ANB), the right–left difference in lateral Ramal inclination; the right–left difference in ramus length (without condyle and gonial angle), the right–left difference in ramus length (with condyle and gonial angle), and the right–left difference in condylar height.

SN.GoGn and FMA were the most reliable indicators in assessing facial vertical growth patterns. An FMA of 25 ± 4° is within a normal range (hypodivergent < 21°, hyperdivergent > 29°). An SN.GoGn of 32 ± 4° is within a normal range (hypodivergent < 28°, hyperdivergent > 36°) (24).

The facial divergence was evaluated with the Sella–Nasion and Gonion–Gnathion angle (*SN*^*G*^*oGn*); the *SN*^*G*^*oGn* angle is an angular measurement that quantifies the inclination of the mandibular base concerning the cranial base. A *SN*^*G*^*oGn* of 32 ± 4° is within a normal range (brachyfacial < 28°, dolichofacial > 36°) (25, 26) found a decrease from 36° to 31° between 6 and 16 years of age.

**Figure 3:**
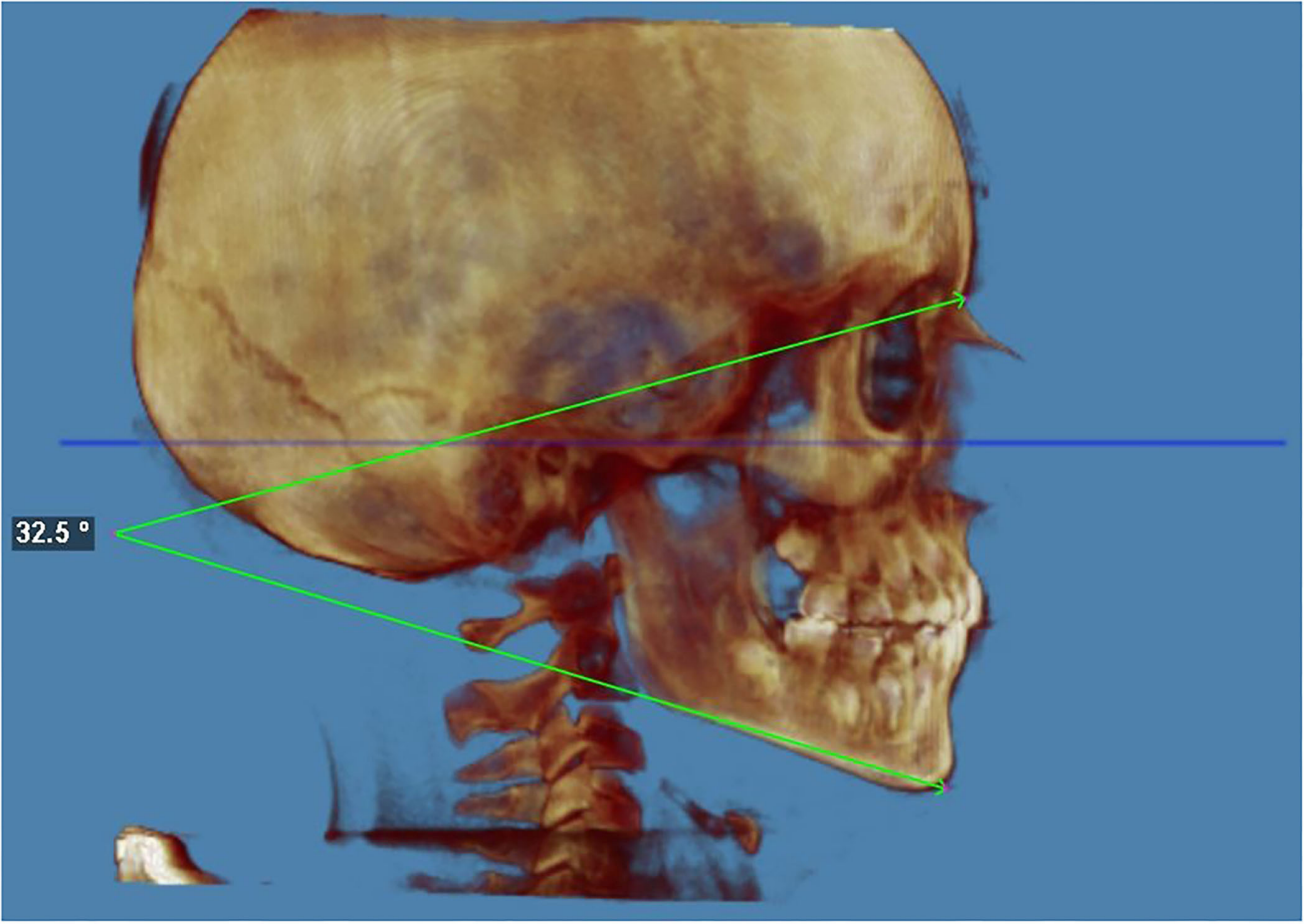
Facial vertical growth pattern at the end of the treatment. The facial divergence was evaluated with the Sella–Nasion and Gonion–Gnathion angle (*SN*^*G*^*oGn*); the *SN*^*G*^*oGn* angle is an angular measurement that quantifies the inclination of the mandibular base about the cranial base. The angle from SN to GoGn was 32.5 (mesofacial) at the end of the treatment. An SN.GoGn of 32 ± 4 degrees is within normal range (hypodivergent < 28° and hyperdivergent > 36°).

The angle from SN to GoGn was 30.1° (mesofacial) at t0 and 32.5° (mesofacial) at t1, with a difference of +2.4° (Figure 3).

The Frankfort horizontal plane–gonion–gnathion angle (*FH*^*G*^*oGn*) is formed by the intersection of the Frankfort horizontal plane (*FH*) and the mandibular plane (*GoGn*). A FMA of 25 ± 5° is within a normal range (hyperdivergent > 30°, hypodivergent < 20°).

**Figure 4:**
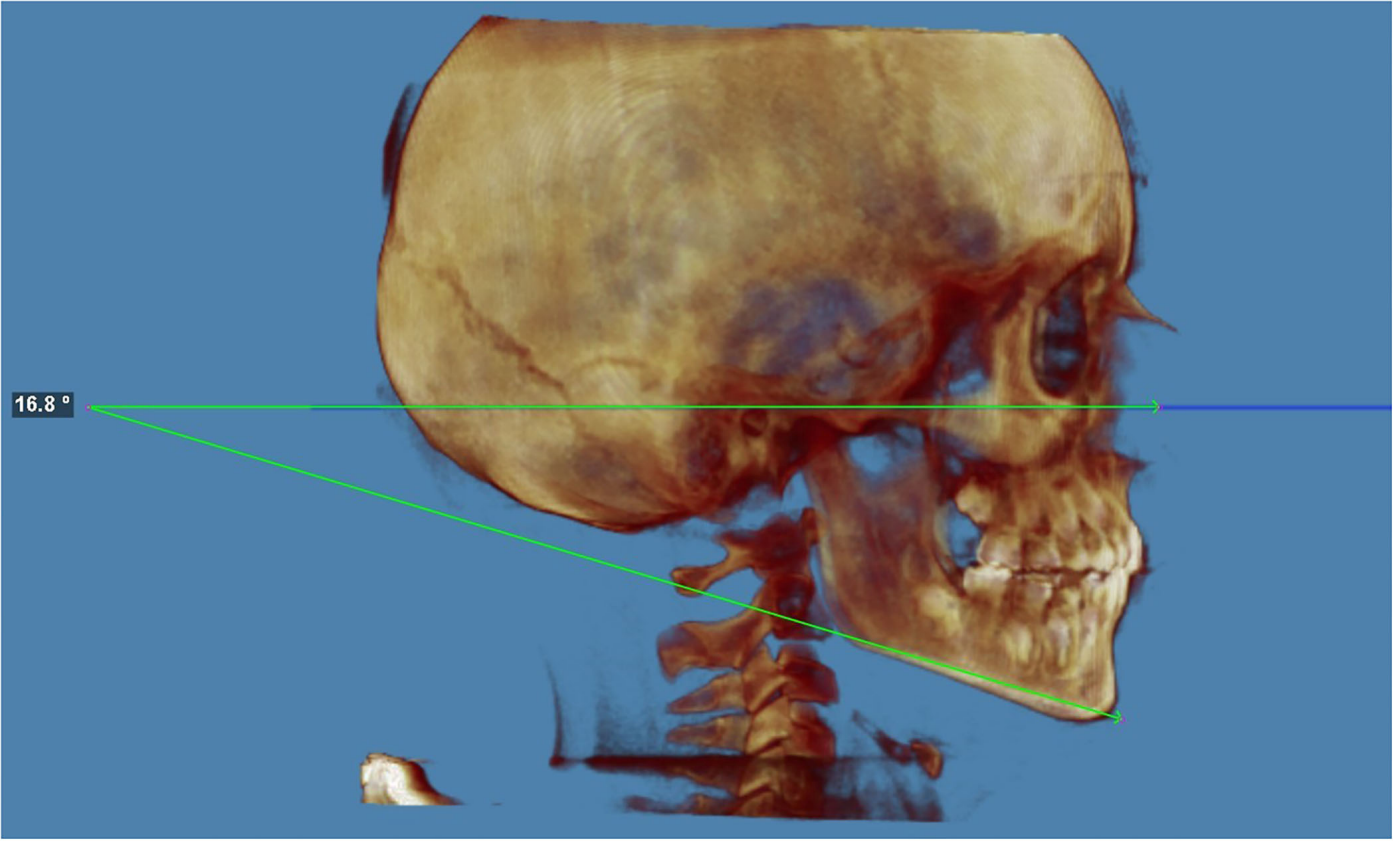
Frankfort-mandibular plane angle (FMA) at the end of the treatment. The FMA is the angle from FH to GoGn. The FMA was 16,8° (hypodivergent) at t1 with a difference of +3.2°. An FMA of 25 ± 4° is within normal range (hypodivergent < 21°, hyperdivergent > 29°).

The FMA was 13.6° (hypodivergent) at t0 and 16.8° (hypodivergent) at t1, with a difference of +3.2° (Figure 4). This result does not differ much from the *SN*^*G*^*oGn*.

**Figure 5:**
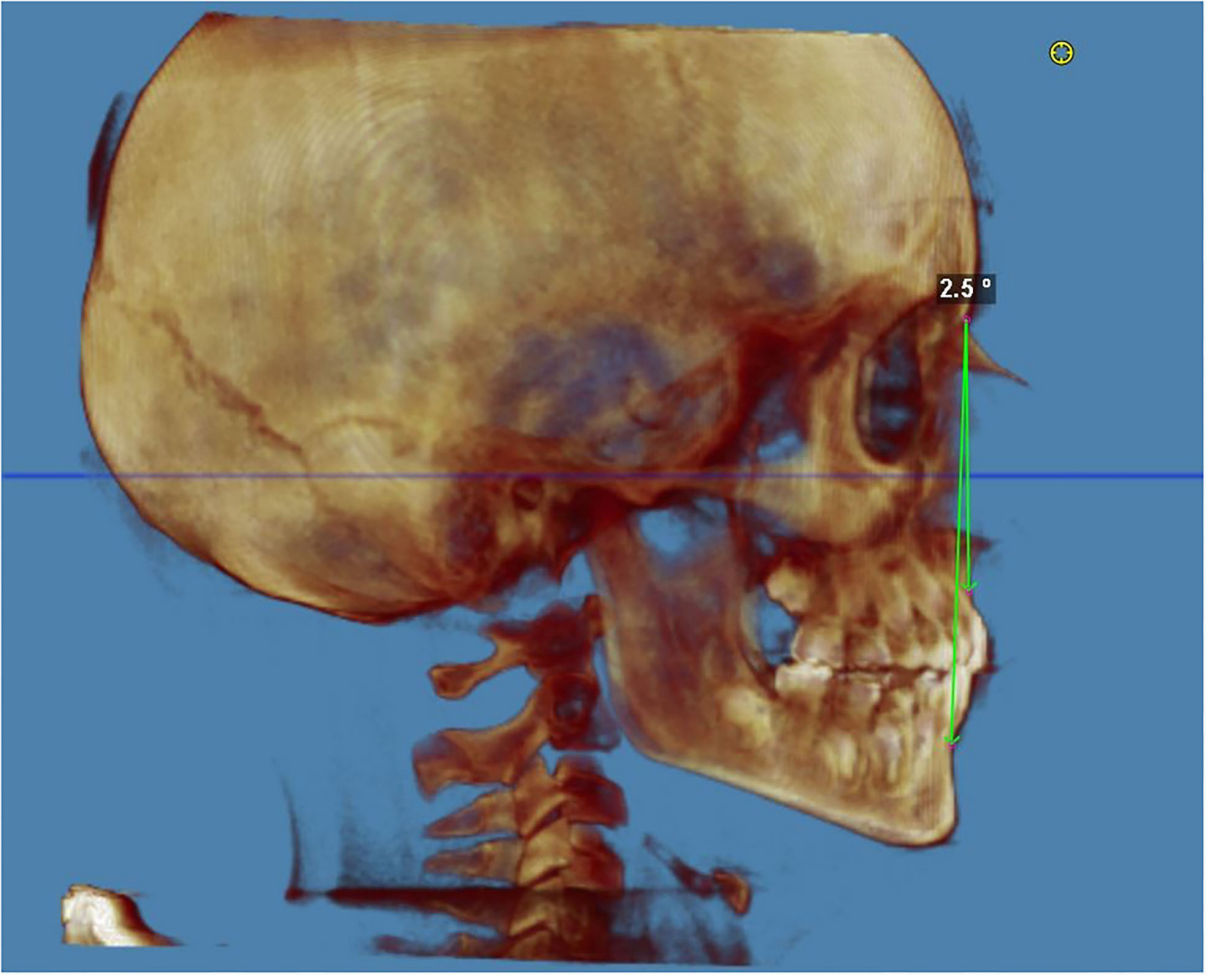
Sagittal facial growth pattern (ANB) at the end of the treatment (LL view). The subspinale–nasion–supramental angle (ANB) indicates the skeletal relationship between the maxilla (at the level of point A) and mandible (at the level of point B). The ANB angle is commonly used to determine the sagittal facial growth pattern in cephalometric analysis, and an ANB of 2 ± 2° is within the normal range (class II > 4°, class III < 0°). The sagittal facial growth pattern (ANB) was 2.5° (class I) at the end of the treatment.

The subspinale–nasion–supramental angle (ANB) indicates the skeletal relationship between the maxilla (at the level of point A) and mandible (at the level of point B). The ANB angle (Figure 5) is commonly used to determine the sagittal facial growth pattern in cephalometric analysis, and an ANB of 2 ± 2° is within a normal range (class II > 4°, class III < 0°).

The sagittal facial growth pattern (ANB) was 0.9° (class I) at T0 and 2.5° (class I) at T1, with a difference of +1.6°.

The inclination of the mandibular ramus was calculated with the angle between Cd post—Go post and FH). The inclination of the right ramus has increased (+2.4°); instead, the inclination of the left ramus has decreased (−1.6°). Also, this result positively affected the re-centring of the menton points toward the MSP.

The height of the mandibular ramus was calculated in different ways: the ramus length without condyle and gonial angle (distance from Copost gopost), the ramus length with condyle and gonial angle (distance from Cdsup to Go inf), and the condylar height (distance from Cdsup to S). In each case, the right side was significantly higher than the left side at t0. At the end of the treatment, the right side was slightly higher than the left side, a sign of more growth on the left side.

The maxillary height was calculated from FH to the occlusal fossa of the maxillary first molar. The right hemimaxilla was slightly higher than the left hemimaxilla at t0. At the end of the treatment, the left hemimaxilla was marginally higher than the right hemimaxilla, a sign of more growth on the left side (+9.8 mm).

The frontal Ramal inclination was calculated with the angle between Cdlat-Golat to MSP. The inclination of the right ramus has decreased (−5.5°); instead, the inclination of the left ramus has increased (+0.5°).

After the treatment, the inclination of the right mandibular ramus has changed more than the left one, as shown in LL and PA view, instead of the inclination of the left mandibular ramus, which has remained relatively unchanged. However, the height evaluation showed that the left ramus had grown more than the right ramus.

The height of the hemi-mandible was evaluated as the distance from the cuspal tip of the mandibular canine to GoGn. The height of the left hemi-mandible was shorter than the right hemi-mandible after the treatment. The pre-treatment height was not evaluated as the canines did not erupt yet.

The intercondylar distance (from the right Cdmed to the left Cdmed) was 74.0 mmm at t0 when the patient was 8 years old. After the treatment, when the patient was 12 years old, the intercondylar distance was 83.3 mm, increasing +by 9.3 mm.

The extracondylar distance was 102.7 mm at t0 and 107.9 mm at t1, increasing +by 5.2 mm.

On SMV view, the cephalometric analysis evaluated the length of the hemimandibular body.

The length of the hemimandibular body was calculated with the distance between the menton point and the gopost point. The right side was slightly longer than the left side at T0.

After the treatment, the length of the right side has slightly increased (+1.0 mm); instead, the length of the left side has significantly increased (+7.5 mm). The left side resulted longer than the right side at the end of the treatment. Also, this result positively affected the re-centring of the menton points toward the MSP.

## Discussion

The purpose of this case report was to evaluate the development and the compensation mechanisms of the mandibular asymmetry in a growing male patient using cone beam computed tomography (CBCT) after treatment with RPE and FR-3 (21).

A low-dose CBCT protocol was used to identify landmarks better and reduce the patient's radiation exposure. The first phase of the treatment consists of using the RPE, which provides a transverse expansion of the maxilla. Maxillary transverse deficiency (MTD or maxillary hypoplasia) is a common problem that affects the normal development of the maxillofacial complex. Therefore, early diagnosis and correction of MTD are essential to achieve a normal transverse skeletal relationship between the maxilla and mandible (21). There are three types of MPS disjunction: RPE (with dental support), miniscrew-assisted rapid palatal expansion (MARPE) with skeletal support, and surgically assisted rapid palatal expansion (SARPE). MARPE and SARPE are used in fused MPS or compromised dental support. The introduction of CBCT in orthodontics allows an accurate analysis of sagittal and vertical growth patterns, which helps decide whether to use conventional (RPE) or unconventional maxillary expansion (MARPE or SARPE). A recent study addressed the potential spontaneous adaptive dentoalveolar compensation of the lower arch during RME (27).

The second phase of the treatment consists of using the FR-3 appliance that promotes mandibular growth in a vertical direction and the growth of the maxilla. Compatible with the present case report, many authors (13, 28) reported that the FR-3 appliance promotes an increase in overjet. The increased ANB angle shows that point A advanced sagittally more than point B; therefore, the maxilla has grown more than the mandible. The left hemi-mandible has grown more than the right one and the height of the left half-maxilla compared to the right one. The increase in bone volume on the non-deviated side is due to the compensation mechanisms that occur when the deviation of the menton is >4 mm (29). A recent study found that RME (with both TB and BB anchorage) could determine a slight opening of the sfeno-occipital synchondrosis, with questionable clinical relevance in terms of promoting maxillary protraction helpful during the functional and orthopedic treatment of class III (30).

In bone specific, the most important vertical bone growth occurs at the left mandibular ramus; therefore, the condyle and the goniac angle on the left side have grown more than on the right side.

The growth of the left hemi-mandible was also confirmed by measuring the inclination of the left ramus external border: the angle with MSP decreased in opposition to the right side, which was slightly increased, proving a strong growth of bone in the transverse direction on the left hemi-mandible, also confirmed by the SMV view. In conclusion, the growing patient with moderate right menton deviation was successfully treated using RPE and FR-3. There was a significant regression of the mandibular asymmetry from moderate grade (4.2 mm) to slight grade (1.3 mm), in addition to the correction of dental characteristics (dental class III and anterior crossbite). These therapeutic goals result from a compensation mechanism: the left hemi-mandible has grown more than the right side, by the literature, which affirms that in case of deviation of the menton >4 mm, the bone volume increases on the non-deviated side.

This treatment protocol is recommended for mandibular asymmetry cases and to use on large samples to better know the effects.

## Data availability statement

The raw data supporting the conclusions of this article will be made available by the authors, without undue reservation.

## Ethics statement

The studies involving human participants were reviewed and approved by University of Chieti G. D'Annunzio. Ethics approval (number 23) was obtained by the hospital's Independent Ethics Committee of Chieti. Written informed consent to participate in this study was provided by the participants' legal guardian/next of kin. Written informed consent was obtained from the patient for publication of this report and any accompanying images.

## Author contributions

FF and MM performed and documented the orthodontic case and exams, MM performed the analysis of the CBCT images. MM conducted a review of the literature and drafted the manuscript. All authors contributed to the article and approved the submitted version.

## Conflict of interest

The authors declare that the research was conducted in the absence of any commercial or financial relationships that could be construed as a potential conflict of interest.

## Publisher's note

All claims expressed in this article are solely those of the authors and do not necessarily represent those of their affiliated organizations, or those of the publisher, the editors and the reviewers. Any product that may be evaluated in this article, or claim that may be made by its manufacturer, is not guaranteed or endorsed by the publisher.
